# Comparative Antennal Transcriptome Analysis of *Phenacoccus solenopsis* and Expression Profiling of Candidate Odorant Receptor Genes

**DOI:** 10.3390/ijms262210901

**Published:** 2025-11-10

**Authors:** Wanying Dong, Ruipeng Chen, Yang Lei, Jun Huang, Yaobin Lu

**Affiliations:** 1 State Key Laboratory for Quality and Safety of Agro-Products, Key Laboratory of Biotechnology in Plant Protection of MOA of China and Zhejiang Province, Institute of Plant Protection and Microbiology, Zhejiang Academy of Agricultural Sciences, Hangzhou 310021, China; dongwy@zaas.ac.cn (W.D.); ly538253@163.com (Y.L.); 2 State Key Laboratory for Biology of Plant Diseases and Insect Pests, Institute of Plant Protection, Chinese Academy of Agricultural Sciences, Beijing 100193, China; m18238756637@163.com; 3 College of Advanced Agricultural Sciences, Zhejiang A&F University, Hangzhou 311300, China; 4 Institute of Bio-Interaction, Xianghu Laboratory, Hangzhou 311231, China

**Keywords:** cotton mealybug, transcriptome, chemosensory genes, sex-biased expression, odorant receptors

## Abstract

Insects rely heavily on olfaction to regulate essential behaviors such as host location, oviposition and mating. The invasive cotton mealybug, *Phenacoccus solenopsis* Tinsley represents a global threat to cotton and numerous cultivated crops. To elucidate the molecular basis of its olfaction mechanisms, we sequenced and assembled antennal transcriptomes from male and female adults using Illumina NovaSeq X Plus technology. Among 13,891 unigenes, 91 chemosensory genes were identified, including 40 odorant receptors, 13 gustatory receptors, 19 ionotropic receptors, 10 odorant-binding proteins, 7 chemosensory proteins, and 2 sensory neuron membrane proteins. Differential expression analysis revealed 6312 genes with significant sex-biased expression between male and female antennae, including 55 chemosensory genes. Phylogenetic analyses further clarified the evolutionary relationships of these chemosensory genes with homologs from other hemipteran species. Notably, validation confirmed that 18 *PsolORs* were male-biased. This comprehensive transcriptomic study establishes a foundation for further functional characterization of pheromone reception and provides valuable candidate genes for dissecting chemoreception mechanisms in *P. solenopsis*.

## 1. Introduction

Insects utilize their highly developed olfactory systems to identify host plants and prey, search for mates and food sources, choose suitable habitats and oviposition sites, and evade predators [1,2]. The antennae function as a crucial olfactory organ in insects, containing diverse types of sensilla that house the dendritic structures of olfactory neurons [3,4]. Insects utilize these varied sensilla to detect intricate chemical signals inside their surroundings [5,6,7]. Diverse chemosensory proteins are essential for insect olfaction, including odorant receptors (ORs), ionotropic receptors (IRs), gustatory receptors (GRs), odorant binding proteins (OBPs), chemosensory proteins (CSPs), and sensory neuron membrane proteins (SNMPs) [8,9,10]. The suggested olfaction mechanism comprises two fundamental processes: (1) Odor molecules penetrate the peripheral olfactory system via sensillum cuticular pores and are conveyed by OBPs to olfactory neuron dendritic membranes, activating ORs. (2) Upon receptor activation, the chemical signal is converted into an electrical signal, which is transmitted and triggers behaviors in the insect [2,11]. Recent advancements in bioinformatics and protein function prediction techniques have markedly expedited research on insect peripheral sensory systems and chemosensory signal transduction [12,13,14]. Antennae transcriptome analyses across diverse insect taxa have produced a substantial amount of valuable data [15].

The cotton mealybug, *Phenacoccus solenopsis* Tinsley (Hemiptera: Pseudococcidae), is a native species of America that was first identified as a cotton pest in the United States in 1991 [16]. Currently widespread worldwide (except Antarctica), subsisting on more than 200 plant species, encompassing agricultural crops and horticulture varieties [17,18]. Phloem feeding by nymphs and females induces leaf deformity, chlorosis, flower/fruit abscission, and growth inhibition. Honeydew accumulation fosters the proliferation of sooty mold, which diminishes photosynthesis and may lead to extensive plant mortality [19]. Currently, *P. solenopsis* continues to pose a significant agricultural threat, especially to cotton cultivation in South Asia [20]. The antennal transcriptome of *P. solenopsis* third instar nymphs and female adults was initially documented in 2018, revealing olfactory-related proteins including 4 ORs, 1 GR, 12 OBPs, 4 CSPs, and 1 SNMP. Furthermore, they conducted a quantitative expression assessment of eight OBPs through several developmental stages and tissues of female adults; nevertheless, sex-biased expression of ORs were undetected [21]. Following the 2018 release of the *P. solenopsis* antennal transcriptome, the functional characterization of its ORs involved in pheromone recognition and sex pheromone-binding proteins has still to be investigated. This indicates potential challenges in identifying sex pheromone receptors in *P. solenopsis*.

In contrast to moth sex pheromones, often composed of straight 10–18 carbon acetates, aldehydes, and alcohols containing 0–3 double bonds [22], scale pheromones are terpenoid products with distinctive backbones [23]. Despite having certain structural similarities, the content of the sex pheromones of each mealybug species differs, with each insect generating unique chemicals [24]. Additionally, the ability to deduce homologous sex pheromone receptors through the sex pheromone receptor evolutionary branch is severely limited due to the paucity of genetic data on the ORs of soft-scale insects, including mealybugs [25]. Therefore, comparative analysis of sex-biased antennal transcriptome expression patterns and diverse OR gene expression in adult *P. solenopsis* could facilitate identification of male-specific OR candidates. This approach might offer vital information for characterizing putative sex pheromone receptors in this species.

Here, we identified candidate chemosensory genes by examining the antennal transcriptomes of *P. solenopsis* males and females. Phylogenetic analysis and expression profiling of the candidate chemosensory genes were also performed to elucidate their potential functions. Furthermore, real-time quantitative PCR (qRT-PCR) was employed to assess the relative expression levels of 30 general OR genes in the antennae of male and female mealybugs. The identification of genes exhibiting male-biased expression establishes a basis for predicting the sex pheromone receptors of *P. solenopsis*. The identification of candidate chemosensory genes paves the path for further investigation into functional evaluations regarding chemoreception association.

## 2. Results

### 2.1. Data Analysis and Functional Annotation

A total of 256.68 million raw reads were generated from antennal transcriptomes of unmated male and female *P. solenopsis* (three biological replicates each). After removing low-quality and N-containing reads, 246.63 million clean reads (36.99 Gb) were retained for downstream analyses. The Q20 scores exceeded 93% across all samples, with GC contents ranging from 32.61 to 35.54% and an average sequencing error rate of only 0.04 (Supplementary file 1: Table S1). Pearson correlation coefficients greater than 0.94 indicated high reproducibility between replicates, confirming that the RNA-seq data were robust and reliable for subsequent analyses (Supplementary file 2: Figure S1A). The BUSCO assessment indicated that a high level of completeness (C: 87%) with a low proportion of missing genes (M: 6.9%), confirming that the reference genome assembly provided a high-quality foundation for the subsequent reference-based transcriptome analysis (Supplementary file 2: Figure S1B). Clean reads were mapped to the *P. solenopsis* reference genome using HISAT2, yielding an average mapping rate of 79.11%, with unique mapping rates ranging from 58.65% to 71.22% (mean 67.31%) and multiple mapping rates from 5.33% to 23.82% (mean 11.81%) (Supplementary file 2: Figure S1C).

**Figure 1:**
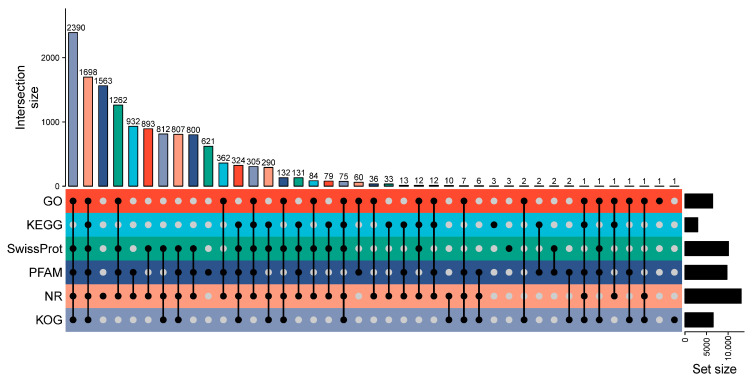
Overview of functional annotation for the *P*. *solenopsis* antennal transcriptome. The UpSet plot visualizes the intersections of unigene annotations across six public databases (NR, Swiss-Prot, PFAM, KOG, GO, and KEGG). The horizontal bar chart on the right shows the total number of unigenes annotated in each individual database. The vertical bar chart at the top shows the size of the intersection for each combination of databases, which is detailed by the connected dots below. A filled black dot indicates that a database is included in the specific intersection set, while an empty gray dot indicates it is not.

Functional annotation was conducted against six major databases. Among the 14,982 unigenes, 13,071 (87.24%) matched entries in NR, 10,100 (67.41%) in Swiss-Prot, 9739 (65.00%) in PFAM, 6560 (43.79%) in KOG, 6443 (43.00%) in GO, and 2979 (19.88%) in KEGG. Overall, 13,770 unigenes (91.91%) were successfully annotated in at least one database, while 1212 (8.09%) remained unannotated (Figure 1, Supplementary file 3: Table S2).

### 2.2. Differential Expression Analysis

To identify differentially expressed genes (DEGs) between male and female antennae, thresholds of *p*_adj_ ≤ 0.05 and |log2FoldChange| ≥ 1.0 were applied using DESeq2 (v.1.20.0). In total 6312 DEGs were detected, comprising 3383 upregulated and 2929 downregulated genes in male antennae relative to female antennae (Supplementary file 4: Figure S2).

### 2.3. GO and KEGG Enrichment

**Figure 2:**
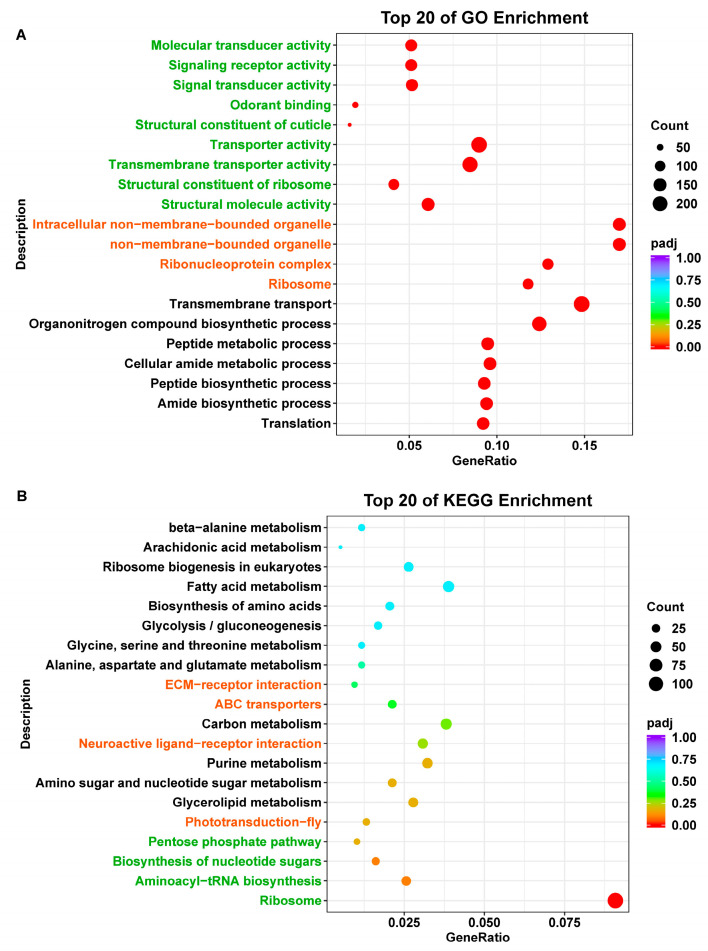
(**A**) GO enrichment bubble plot of the antennal transcriptome of *P. solenopsis* (male vs. female). Dot plot showing the top 20 significantly enriched GO terms from the Biological Processes (BPs), Molecular Functions (MFs), and Cellular Components (CCs). The text color represents the GO category (BP, black; MF, green; CC, orange). The complete list of enriched GO terms is available in Supplementary file 5: Table S3. (**B**) KEGG enrichment bubble plot of the antennal transcriptome of *P. solenopsis* (male vs. female). Dot plot showing the top 20 significantly enriched KEGG pathways from the Metabolism (M), Environmental Information Processing (EIP), and Organismal Systems (OSs). The text color represents the KEGG category (M, black; EIP, orange; OS, green). The complete list of enriched KEGG pathways is available in Supplementary file 6: Table S4.

Gene Ontology (GO) enrichment analysis yielded 685 significantly enriched terms. The top 20 terms across the three main GO categories—Biological Processes (BPs), Molecular Functions (MFs), and Cellular Components (CCs)—are shown in Figure 2A. Within BP, the most represented subcategories were “transmembrane transport”, “organonitrogen compound biosynthetic process”, and “cellular amide metabolic process”. Within MF, the predominant terms were “transporter activity”, “transmembrane transporter activity”, and “structural molecule activity”, while “non-membrane-bounded organelle”, “intracellular non-membrane-bounded organelle”, and “ribonucleoprotein complex” were the most enriched in CC. In total, 3583 of the 20,504 GO-annotated genes were differently expressed.

KEGG enrichment analysis further classified DEGs into three main categories Metabolism, Environmental Information Processing, and Organismal Systems. The Metabolism category included nine subpathways, such as glycerolipid metabolism, amino sugar and nucleotide sugar metabolism, purine metabolism, and carbon metabolism, none of which contained DEGs in *P. solenopsis* antennae. Similarly, the Environmental Information Processing category (including phototransduction—fly, neuroactive ligand–receptor interaction, ABC transporters, and ECM–receptor interaction) showed no differentially expressed genes. Within Organismal Systems, four pathways were represented—ribosome, aminoacyl–tRNA biosynthesis, biosynthesis of nucleotide sugars, and pentose phosphate pathway—with only 124 DEGs annotated under the ribosome subcategory (Figure 2B).

### 2.4. Candidate ORs in P. solenopsis

Transcriptome analysis identified 40 putative ORs transcripts across male and female antennae. Among these, 21 PsolORs contained full-length open reading frames (ORFs) encoding proteins with six to seven transmembrane domains (TMDs). Comparative analysis revealed that these PsolORs shared 22–95% amino acid identity with ORs from other insects (Supplementary file 7: Table S5-1).

**Figure 3:**
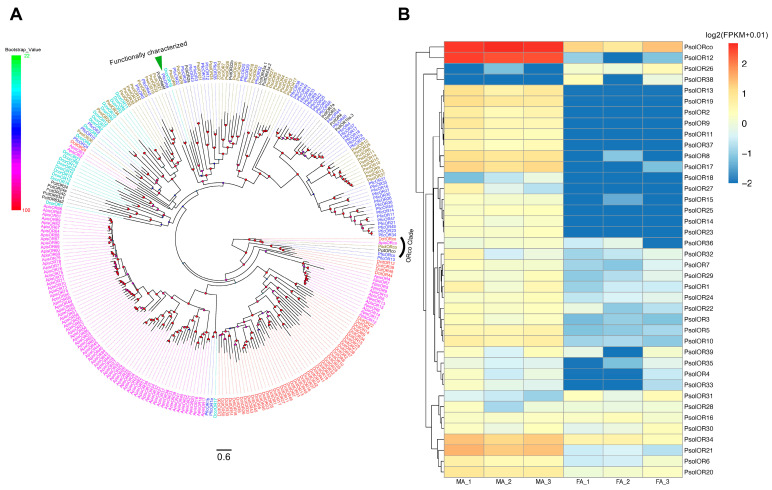
Analyses of candidate odorant receptors (ORs). (**A**) Phylogenetic tree of candidate *P. solenopsis* ORs and other hemipteran ORs. The phylogenetic tree was rooted using ORco orthologues, and bootstrap values are shown. Psol, *Phenacoccus solenopsis* (brown); Pfic, *Planococcus ficus* (blue); Pcit, *Planococcus citri* (black); Apis, *Acyrthosiphon pisum* (magentas); Dcor, *Drosicha corpulenta* (cyans); Dcit, *Diaphorina citri* (red). The GenBank accession numbers/references used in this analysis are listed in Supplementary file 8: Table S6. (**B**) Heatmap of PsolOR gene expression levels in the male and female antennae. The data were standardized as follows: log2(FPKM + 0.01). FA: female antennae, MA: male antennae.

Phylogenetic analysis was performed using OR sequences from *P. solenopsis* and five other hemipterans–*Planococcus citri*, *Planococcus ficus*, *Drosicha corpulenta*, *Acyrthosiphon pisum*, and *Diaphorina citri* (Figure 3A). The conserved co-receptor (ORco) from *P. solenopsis* (designated PsolORco) clustered with DcitORco, ApisORco, PcitORco, and PficORco. Approximately three-quarters of PsolORs were dispersed throughout the phylogenetic tree, indicating broad evolutionary relationships with other hemipteran species. A *P. solenopsis* species-specific clade consisting of ten members (PsolOR8, 9, 10, 11, 12, 13, 14, 15, 25 and 37) exhibited low sequence identity to other hemipteran ORs, suggesting potential species-specific functions.

Of the 40 PsolORs identified, PsolOR38 was absent in male antennae (readcount = 0), while only 29 PsolORs were expressed in female antennae. The co-receptor ORco exhibited the highest expression in both sexes (male antennae: 426.31 FPKM; female antennae: 15.93 FPKM), followed by PsolOR12, PsolOR21 and PsolOR34 (Figure 3B, Supplementary file 7: Table S5-1). In total, 30 PsolORs displayed significant sex-biased expression (*p*_adj_ < 0.05, |log2FoldChange| > 1), with 28 being male-biased and 2 female-biased.

### 2.5. Candidate GRs in P. solenopsis

**Figure 4:**
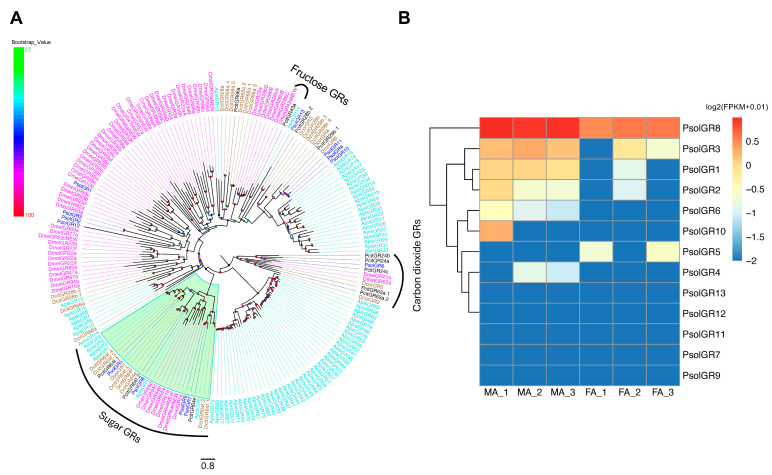
Analyses of candidate gustatory receptors (GRs). (**A**) Phylogenetic tree of candidate *P. solenopsis* GRs and other insect GRs. The phylogenetic tree was rooted using sugar GR orthologues, and bootstrap values are shown. The green highlighted part relates to “Sugar receptor.” Psol, *Phenacoccus solenopsis* (brown); Pcit, *Planococcus citri* (cyans); Apis, *Acyrthosiphon pisum* (magentas); Dcor, *Drosicha corpulenta* (black); Dcit, *Diaphorina citri* (blue). The GenBank accession numbers/references used in this analysis are listed in Supplementary file 8: Table S6. (**B**) Heatmap of PsolGR gene expression levels in the male and female antennae. The data were standardized as follows: log2(FPKM + 0.01). FA: female antennae, MA: male antennae.

Thirteen GR genes were identified in the antennal transcriptomes. Most PsolGRs were partial sequences, with only three encoding full-length peptides. All GR proteins exhibited the typical seven TMDs, characterized by intracellular N-termini and an extracellular C-terminus (Supplementary file 7: Table S5-2). Phylogenetic analysis incorporating GR sequences from four hemipteran species and *Drosophila melanogaster* showed that four PsolGRs (PsolGR5, PsolGR7, PsolGR8 and PsolGR9) were closely related to the sugar receptors ApisGR1-6 of *A. pisum*, which is functional in sugar detection. PsolGR6 clustered with the carbon dioxide receptor subfamily. However, no homologs of fructose receptors were identified in the *P. solenopsis* transcriptome (Figure 4A).

Eight PsolGRs (PsolGR1-6, PsolGR8, and PsolGR10) were detected in both sexes, while the remaining genes were expressed in non-antennal tissues. Among them, PsolGR1, PsolGR3, and PsolGR8 exhibited higher expression in male antennae, whereas PsolGR2, PsolGR4, PsolGR5, PsolGR6, and PsolGR10 showed no significant sex-biased differences (Figure 4B).

### 2.6. Candidate IRs in P. solenopsis

Nineteen putative ionotropic receptor (IR) transcripts were identified, five of which contained full-length ORFs with three to five TMDs and lengths ranging from 98 to 1611 amino acids (Supplementary file 7: Table S5-3). Phylogenetic analysis incorporating IR sequences from *D. melanogaster*, *Aphis glycines*, *A. pisum*, and *P. solenopsis* revealed that PsolIR8a, PsolIR25a and PsolIR93a clustered within a conserved co-receptor subfamily. However, the canonical co-receptor IR76b was absent from *P. solenopsis* assemblies. Eight PsolIRs (IR6, IR8, IR9, IR21a, IR40a, IR68a, IR68a.1 and IR323) grouped with presumed “antennal IR” orthologues, while the remaining PsolIRs (IR1-5, IR7, IR100a and IR325) belonged to a “divergent IR” clade.

**Figure 5:**
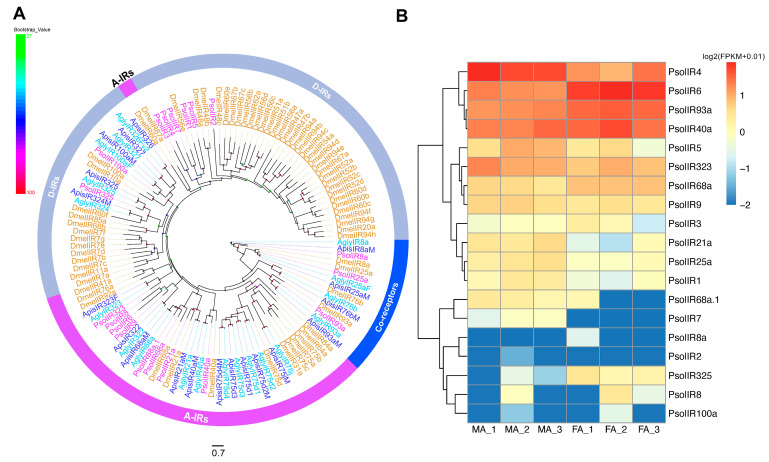
Analyses of candidate ionotropic receptors (IRs). (**A**) Phylogenetic tree of candidate *P. solenopsis* IRs and other insect IRs. The phylogenetic tree was rooted using IR8a/IR25a/IR93a orthologues, and bootstrap values are shown. The outer ring annotates the gene subfamily classification (A-IRs, antennal IRs; D-IRs, divergent IRs). Psol, *Phenacoccus solenopsis* (magentas); Dmel, *Drosophila melanogaster* (brown); Agly, *Aphis glycines* (wathet); Apis, *Acyrthosiphon pisum* (blue). The GenBank accession numbers/references used in this analysis are listed in Supplementary file 8: Table S6. (**B**) Heatmap of PsolIR gene expression levels in the male and female antennae. The data were standardized as follows: log2(FPKM + 0.01). FA: female antennae, MA: male antennae.

Based on FPKM values, PsolIR6 showed the highest expression (MA: 17.62, FA: 68.50, mean FPKM), followed by PsolIR4 (male: 59.25, female: 16.55). PsolIR2 and PsolIR7 were expressed exclusively in male antennae, whereas PsolIR325 was predominantly expressed in females. Seven IRs (IR1, IR4, IR5, IR6, IR21a, IR25a and IR325) exhibited significant sex-biased expression between males and females (Figure 5B).

### 2.7. Candidate OBPs in P. solenopsis

**Figure 6:**
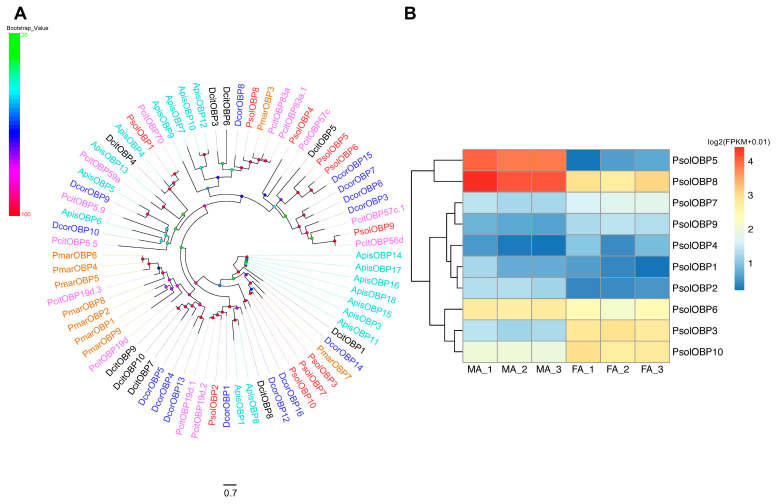
Analyses of candidate odorant-binding proteins (OBPs). (**A**) Phylogenetic tree of candidate *P. solenopsis* OBPs and other hemipteran OBPs. Bootstrap values are shown on the left. Psol, *Phenacoccus solenopsis* (red); Pcit, *Planococcus citri* (magentas); Pmar, *Paracoccus marginatus* (brown); Apis, *Acyrthosiphon pisum* (cyans); Dcor, *Drosicha corpulenta* (blue); Dcit, *Diaphorina citri* (black). The GenBank accession numbers/references used in this analysis are listed in Supplementary file 8: Table S6. (**B**) Heatmap of PsolOBP gene expression levels in the male and female antennae. The data were standardized as follows: log2(FPKM + 0.01). FA: female antennae, MA: male antennae.

A total of ten transcripts encoding candidate OBPs were identified in *P. solenopsis*. Eight sequences were complete, encoding proteins ranging from 135 to 199 amino acids, and seven contained predicted N-terminal signal peptides, with the exception of PsolOBP7 (Supplementary file 7: Table S5-4). Phylogenetic analysis using orthologous OBP sequences from five hemipteran species revealed that the PsolOBPs were distributed across distinct clades. All ten PsolOBPs were classified within the classical OBP subfamily, characterized by six conserved cysteines residues arranged in the typical motif C1-X15-39-C2-X3-C3-X21-24-C4-X7-12-C5-X8-C6 (Figure 6A, Supplementary file 9: Figure S3).

Expression profiling showed that all ten PsolOBPs were present in both males and female antennae but displayed sex-biased expression. PsolOBP1, PsolOBP2, PsolOBP5, PsolOBP6, and PsolOBP8 were significantly upregulated in males, whereas PsolOBP3, PsolOBP4, PsolOBP7, PsolOBP9, and PsolOBP10 were more highly expressed in females (Figure 6B).

### 2.8. Candidate CSPs in P. solenopsis

**Figure 7:**
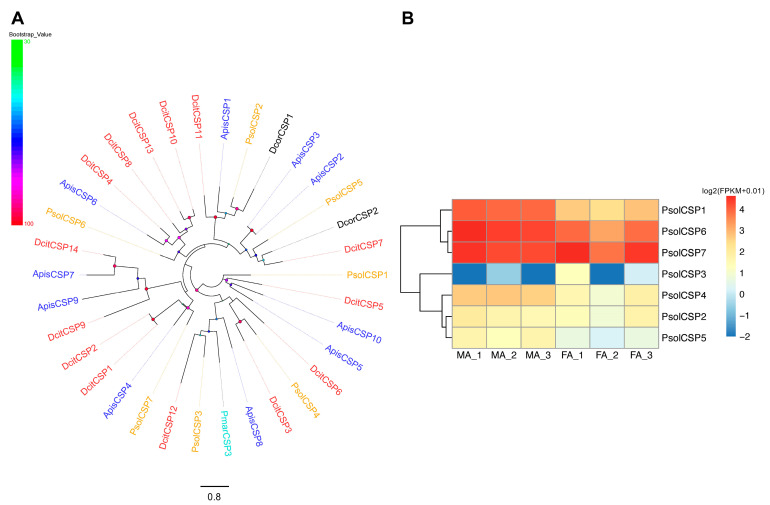
Analyses of candidate odorant receptors (CSPs). (**A**) Phylogenetic tree of candidate *P. solenopsis* CSPs and other hemipteran CSPs. Bootstrap values are shown on the left. Psol, *Phenacoccus solenopsis* (brown); Pmar, *Paracoccus marginatus* (cyans); Apis, *Acyrthosiphon pisum* (blue); Dcor, *Drosicha corpulenta* (black); Dcit, *Diaphorina citri* (red). The GenBank accession numbers/references used in this analysis are listed in Supplementary file 8: Table S6. (**B**) Heatmap of PsolCSP gene expression levels in the male and female antennae. The data were standardized as follows: log2(FPKM + 0.01). FA: female antennae, MA: male antennae.

Seven CSPs were identified from the antennae transcriptomes of *P. solenopsis*. Five of these (PsolCSPs) encoded full-length ORFs for proteins comprising 111 and 136 amino acids, all predicted to possess N-terminal signal peptides (Supplementary file 7: Table S5-5). All CSPs had four conserved cysteine residues (Supplementary file 10: Figure S4). A phylogenetic tree constructed using CSP sequences from *P. solenopsis*, *P. marginatus*, *A. pisum*, *D. corpulenta*, and *D. citri* revealed that PsolCSPs were distributed among distinct branches, showing close homology with those of *D. corpulenta* and *D. citri* (Figure 7A).

All PsolCSPs were expressed in both male and female antennae. PsolCSP2 and PsolCSP7 showed no significant sex-biased expression. Differential expression analysis indicated that PsolCSP3 was significantly upregulated in female antennae, while PsolCSP1, PsolCSP4, PsolCSP5, and PsolCSP6 were expressed at higher levels in male antennae (*p* < 0.05) (Figure 7B).

### 2.9. Candidate SNMPs in P. solenopsis

**Figure 8:**
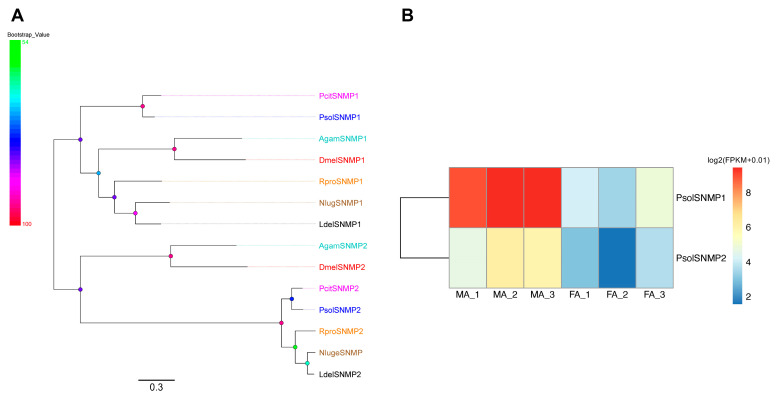
Analyses of candidate sensory neuron membrane proteins (SNMPs). (**A**) Phylogenetic tree of candidate *P. solenopsis* SNMPs and other insect SNMPs. Bootstrap values are shown on the left. Psol, *Phenacoccus solenopsis* (blue); Pcit, *Planococcus citri* (magentas); Dmel, *Drosophila melanogaster* (red); Agam, *Anopheles gambiae* (cyans); Nlug, *Nilaparvata lugens* (brown); Ldel, *Lycorma delicatula* (black); Rpro, *Rhodnius prolixus* (orange). The GenBank accession numbers/references used in this analysis are listed in Supplementary file 8: Table S6. (**B**) Heatmap of PsolSNMP gene expression levels in the male and female antennae. The data were standardized as follows: log2(FPKM + 0.01). FA: female antennae, MA: male antennae.

Two SNMPs were identified from *P. solenopsis* antennae transcriptomes, both encoding full-length ORFs and possessing a single TMD (Supplementary file 7: Table S5-6). Phylogenetic analysis incorporating PsolSNMPs and orthologs from *P. citri*, *Anopheles gambiae*, *D. melanogaster*, *Nilaparvata lugens*, *Lycorma delicatula*, and *Rhodnius prolixus* revealed that both PsolSNMPs clustered with single-copy orthologs from *P. citri* (Figure 8A). Expression analysis further showed that both PsolSNMPs were significantly upregulated in male antennae (Figure 8B).

### 2.10. Expression Levels of PsolOR Genes by qRT-PCR

**Figure 9:**
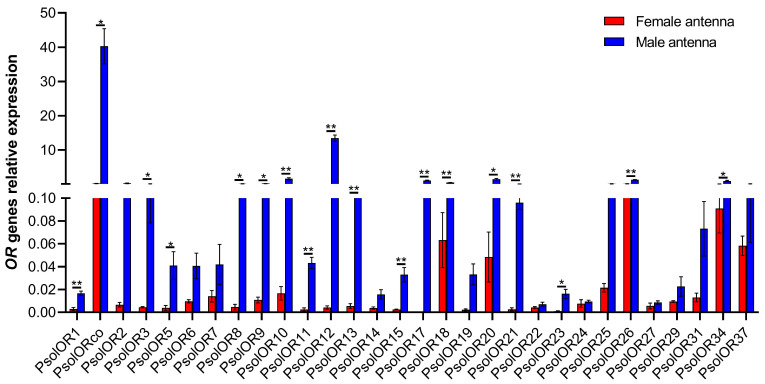
Analysis of differentially expressed odorant receptors in the antennae of male and female *P. solenopsis*. Asterisks indicate significant differences (* *p* < 0.05, ** *p* < 0.01), independent *t*-test. Data are shown as the mean ± standard error.

To validate the differential expression of *PsolOR* genes between male and female antennae, qRT-PCR was performed for all 30 *PsolOR* DEGs. The expression profiles of these genes are presented in Figure 9. Independent sample *t*-test confirmed that all 30 *PsolORs* were upregulated in the male antennae, while expression levels in female antennae were markedly lower. Among these, 18 *PsolORs*-notably *PsolOR1*, *PsolOR10*, *PsolOR11*, *PsolOR12*, *PsolOR13*, *PsolOR15*, *PsolOR17*, *PsolOR18*, *PsolOR21*, and *PsolOR26*-exhibited significantly higher expression in males. The remaining *PsolORs* showed no significant sex-specific differences in transcript abundance. The co-receptor gene *ORco* displayed consistently high expression in both male and female antennae.

## 3. Discussion

*Phenacoccus solenopsis*, commonly known as the cotton mealybug, is a major pest of economically important crops, particularly cotton, across Asian cotton-producing regions [20]. Chemical cues play a central role in mediating insect behaviors, including host location, mate recognition, and avoidance of parasitoids [26,27,28]. These processes depend heavily on chemosensory proteins. In this study, we analyzed the antennal transcriptomes of *P. solenopsis* to identify chemosensory genes and to elucidate mechanisms underlying chemical communication between males and females, as well as interactions involving host plants, ants, other hemipterans, and natural enemies.

Illumina-based antennal transcriptome sequencing of adult males and females produced high-quality data with excellent Q20/Q30 values and strong correlations among biological replicates, ensuring reliable downstream analyses [29]. The average mapping rate of 79.11% indicated efficient alignment to the reference genome, while unmapped reads may represent antenna-specific or novel transcripts absent from current annotations. Functional annotation revealed marked sex-specific differences: a greater number of male-biased differentially expressed genes (DEGs) suggests higher functional complexity in male antennae, consistent with morphological evidence of denser sensilla [30]. Conversely, enrichment of the ribosome pathway in females may indicate greater protein synthesis demands, whereas other key metabolic pathways appeared conserved between sexes. Future analyses could employ redundancy reduction tools, such as REVIGO, to refine enriched GO terms and highlight the most relevant non-redundant biological processes.

A total of 91 putative chemosensory genes were in *P. solenopsis*, comparable to those reported in other hemipteran antennae transcriptomes—such as *Schlechtendalia chinensis* (79) [31]—but fewer than in *Tropidothorax elegans* (179) [32], *Halyomorpha halys* (238) [33], *Graphosoma rubrolineatum* (185) [34], and *A. pisum* (212) [35]. These differences likely reflect species-specific adaptation to diverse host plants, leading to the diversification of chemosensory genes during evolution. The number of genes identified here substantially exceeds the 22 olfactory-related genes previously reported by Nie [21], possibly due to differences in tissue preparation, sequencing depth, or bioinformatics pipelines.

Odorant receptors are key determinants of insect olfaction specificity and sensitivity [1]. We identified 40 OR genes, fewer than in *P. ficus* (50) [25], *A. linedatus* (88) [36], *H. halys* (138) [37], or *A. pisum* (87) [35]. This lower count may reflect sequencing depth or methodological differences, as genes with low expression are often difficult to detect. Several PsolORs formed species-specific clades, suggesting potential roles in recognizing volatiles from hosts, mates, or oviposition sites.

Interestingly, although the antennal transcriptome included 40 *PsolORs*, only 29 were expressed in female antennae, and all exhibited relatively low expression compared with males. This pattern may reflect to flight capacity: since female *P. solenopsis* are flightless, they likely depend less on olfaction, whereas males—being capable of flight—rely on airborne pheromone to locate mates. This phenomenon is consistent with the fact that the insect olfactory system quickly developed alongside the capacity to fly [38]. The higher abundance of *PsolOR* transcripts in males supports this viewpoint. During mate searching, males detect female-emitted sex pheromones, a process requiring highly sensitive receptors in male antennae [39,40].

Sex-biased expression analyses showed that most *PsolOR* genes were male-enriched, consistent with DEG analyses based on FPKM values, except *PsolOR26* and *PsolOR31*. Although transcriptome data suggested these two genes were upregulated in females, qRT-PCR validation revealed no significant sex difference. This inconsistency could stem from biological or technical variation, including limited statistical power due to the small number of biological replicates (*n* = 3), a constraint imposed by the difficulty of obtaining sufficient antennal tissue from this minute species. While our dataset effectively captured robust DEGs, increasing replication in future studies will help detect subtler transcriptional differences. Although sex pheromone receptor genes have not yet been characterized in *P. solenopsis*, recent studies in *P. ficus* and *Orthaga achatina* have identified overexpressed ORs in males and confirmed their functions via heterologous expression [25,41]. Screening male-enriched ORs and functionally characterizing candidate pheromone receptors will be essential to elucidate molecular mechanism underlying sex pheromone perception in *P. solenopsis*.

Thirteen GR genes were identified in *P. solenopsis* antennae. These likely represent only a subset of the total GR repertoire, as certain members may be restricted to gustatory organs, such as the labium and legs. The number of PsolGRs is lower than in most hemipteran antennal transcriptomes, possibly reflecting limited antennal expression or species-specific sensory specialization [33,34]. Several GRs may function as contact or taste receptors [42]. Four PsolGRs (PsolGR5, PsolGR7, PsolGR8 and PsolGR9) showed homology to the sugar receptor family (ApisGR1-6) in *A. pisum*. PsolGR6 showed homology to the carbon dioxide receptor DmelGR21a and DmelGR63a in *D. melanogaster*. However, limited genomic information on soft-scale insects constrains broader comparative analyses. The elevated expression of PsolGR5 in females suggests a key role in gustatory perception and feeding behavior. Additional validation through qRT-PCR, in situ hybridization, and functional assays will clarify the roles of these GRs.

Nineteen IR genes were identified in *P. solenopsis* antennae, identical in number to those reported in *A. pisum* (19) and *A. glycines* (19) [35], but fewer than in *H. halys* (24) [37] or *G. rubrolineatum* (23) [34]. This variation may reflect ecological adaptation or differential tissue expression. Based on the established classification of DmelIRs [43], the PsolIRs clustered into three groups: eight “antennal IRs” that were highly expressed in antennae and likely involved in sensing acids or polyamines [44,45]; eight “divergent IRs” with low antennal expression, possibly associated with gustation [46]; and three conserved “co-receptor IRs” (PsolIR8a, PsolIR25a, and PsolIR93a). Notably, IR76b was absent. Co-receptors typically form complexes with other IRs, facilitating broad chemosensory functions [47,48,49]. However, functional characterization of IRs in hemipterans remains limited, underscoring the need for further studies.

OBPs and CSPs play essential roles in the transport of hydrophobic odorants—the initial step of olfactory signal transduction [9]. We identified 10 PsolOBPs and 7 PsolCSPs in *P. solenopsis* antennae. The numbers of these gene families vary widely among hemipterans: *Riptortus pedestris* possesses 49 OBPs and 25 CSPs [50], *H. halys* has 44 OBPs and 17 CSPs [37], *Adelphocoris suturalis* has 16 OBPs and 8 CSPs [51], and *A. lineolatus* has 17 OBPs and 10 CSPs [52]. All 10 *PsolOBPs* exhibited significant sex-biased expression, with five upregulated in males and five in females. Comparable sex-biased patterns were also reported in *R. pedestris*, where three OBPs were male-biased and nine were female-biased [50]. Future comparative phylogenetic studies incorporating OBPs from a wider range of hemipteran species, following the framework of Venthur et al. (2014) [53], will be valuable to determine the precise orthologous relationships and evolutionary history of these *P. solenopsis* OBPs. In contrast, CSPs are typically fewer in number across hemipterans [33,35,37] and exhibit extensive evolutionary divergence, likely reflecting their broad physiological versatility beyond olfaction.

The SNMP family in insects is typically categorized into three subfamilies: SNMP1, SNMP2, and SNMP3 [54,55]. Our analysis of the *P. solenopsis* antennal transcriptome identified two of these—PsolSNMP1 and PsolSNMP2—representing the SNMP1 and SNMP2 subfamilies, respectively. The absence of SNMP3, which is primarily expressed in lepidopteran midguts and is generally absent from antennal tissues, is consistent with the tissue-specific nature of our transcriptome data [55]. Notably, PsolSNMP1 was phylogenetically assigned as an ortholog of DmelSNMP1, a receptor essential for the detection of the sex pheromone cis-vaccenyl acetate in *D. melanogaster* [56]. This conserved orthology strongly suggests that PsolSNMP1 may play a parallel role in the perception of sex pheromones in *P. solenopsis*. The specific and biased expression of both PsolSNMPs in male antennae further supports their potential involvement in conspecific chemical communication. Therefore, the functional characterization of these SNMPs, particularly PsolSNMP1, represents a critical next step. Future studies employing techniques such as RNA interference (RNAi) or heterologous expression systems will be essential to definitively establish their role in pheromone reception and to elucidate the underlying molecular mechanisms in *P. solenopsis*.

## 4. Materials and Methods

### 4.1. Insect Rearing and Tissue Collection

Specimens of *Phenacoccus solenopsis* were collected from *Portulaca grandiflora* (Hook.) (Caryophyllales: Portulacaceae) plants in the suburbs of Xiaoshan District, Hangzhou City, Zhejiang Province, China, and raised on sprouting potato tubers in cages for five successive generations under controlled settings (27 ± 1 °C, 60–80% RH, and 16 light: 8 dark hours) within the climate chamber. After eclosion, males and females were underwent separation, their antennae were removed and flash-frozen in liquid nitrogen before −80 °C storage. Triplicate samples were obtained from males and females, each containing 300 pairs of antennae.

### 4.2. cDNA Library Construction and Sequencing

As directed by the manufacturer, total RNA was extracted from the antenna using RNAprep Pure Micro Kit (TIANGEN Biotech (Beijing) Co., Ltd., Beijing, China). An Agilent 2100 Bioanalyzer (Agilent Technologies, Palo Alto, CA, USA) and a NanoDrop ND-2000 spectrophotometer (NanoDrop Products, Wilmington, DE, USA) were used to confirm the quality of the total RNA. A SMARTer^®^ Ultra^®^ Low RNA kit (Takara, CA, USA) was used to generate a SMARTer library for further sequencing. At the Novogene Bioinformatics Institute (Beijing, China), RNA sequencing libraries were constructed and sequenced using a paired-end (PE150 bp) method on the Illumina NovaSeq X Plus (Illumina, San Diego, CA, USA). Raw sequences (accession number: PRJNA1314049) were deposited in the Short Read Archive database (the National Center for Biotechnology Information, NCBI; https://www.ncbi.nlm.nih.gov/sra, accessed on 25 August 2025).

### 4.3. RNA-Seq Data Analysis

Clean 150 bp reads were obtained. Adapter-containing, N base-containing, and low-quality reads were eliminated from the raw data to produce clean reads. The reference genome and annotation files were acquired from Insectbase 2.0 [57]. Genome completeness was quantified using Benchmarking Universal Single Copy Orthologs (BUSCO v4.0.5) [58] (parameters: metazoa_odb10). Hisat2 (v2.0.5) built the reference genome index and aligned the reads [59]. StringTie (v1.3.3b) assembled novel transcripts from the unmapped reads [60]. The unigenes were annotated by BLAST (http://www.ncbi.nlm.nih.gov/BLAST/, accessed on 5 April 2025)-comparing them to databases of the Swiss-Prot protein, the Protein Families (PFAM), the NCBI non-redundant protein (NR), the Eukaryotic Orthologous Groups of protein (KOG), and the Kyoto Encyclopedia of Genes and Genomes (KEGG) with the BLASTx program at *E* ≤ 1 × 10^−5^ [61]. The Blast2GO (v.2.5.0) software was used to import the BLAST results for gene ontology (GO) annotation [62]. GO and KEGG pathway enrichment analyses were carried out using the ClusterProfiler (3.8.1) to identify functional categories and metabolic pathways significantly over-represented in the set of DEGs. All assembled unigenes were annotated against the GO and KEGG databases to define the transcriptomic background. A hypergeometric test was then applied to the DEGs to identify GO terms and KEGG pathways that were significantly enriched (*p* < 0.05) compared to this background [63].

### 4.4. Differentially Expressed Gene Analysis

Using RSEM software, the fragment per kilobase of transcript per million mapped reads (FPKM) value was computed for each transcription region in order to characterize its transcription level [64]. The R package heatmap (v.1.0.12) was used to create the gene expression level heatmap visualizations. DESeq2 was used to assess differential transcriptions based on the negative binomial distribution [65]. Using the R package ggplot2 (v.3.3.5), a volcano plot of DEG was produced, with |log2FoldChange| ≥ 1.0 and *p*-value ≤ 0.05 as threshold.

### 4.5. Identification of Chemosensory Genes

Chemosensory genes (ORs, IRs, GRs, OBPs, CSPs, and SNMPs) were identified through transcriptome assembly and GO annotations [66]. From the functional annotations, candidate genes were isolated based on keywords relevant to chemosensory analysis. With chemosensory representatives from Hemiptera as queries, tBLASTn searches were conducted to identify candidate unigenes, using *E* ≤ 1 × 10^−5^. To further verify all candidate genes, we manually aligned transcripts against all known proteins using the BLASTX NCBI database. Start and termination codons are present in the full-length transcripts. ORF Finder (https://www.ncbi.nlm.nih.gov/orffinder, accessed on 9 June 2025) was used to manually verify the open reading frames (ORFs) of candidate chemosensory genes. The SignalP 6.0 server’s default settings (https://services.healthtech.dtu.dk/services/SignalP-6.0/, accessed on 11 June 2025) were used to predict N-terminal signal peptides from OBPs and CSPs [67]. To predict the transmembrane domains of candidate ORs, IRs, GRs, and SNMPs, DeepTMHMM web server (https://dtu.biolib.com/DeepTMHMM, accessed on 12 June 2025) under default settings was utilized, which provides deterministic results [68]. The nucleotide sequences of candidate chemosensory genes were converted to amino-acid sequences using the ExPASy (Expert Protein Analysis System) server version (https://web.expasy.org/translate/, accessed on 13 June 2025) [69]. The amino acid sequences of chemosensory genes identified in *P. solenopsis* are listed in Supplementary file 11: Table S7.

### 4.6. Sequencing Alignment and Phylogenetic Analysis

MAFFT (https://www.ebi.ac.uk/Tools/msa/mafft/, accessed on 13 August 2025) was used to align amino acid sequences after redundancy was eliminated [70]. The maximum likelihood tree construction with IQ-TREE (v.2.0.7) under the best-fit substitution model (selected by ModelFinder) [71]. Using a bootstrap approach with 1000 replicates, node support was evaluated. The phylogenetic trees were viewed and edited using FigTree v.1.4.4 (http://tree.bio.ed.ac.uk/software/figtree, accessed on 25 August 2025).

### 4.7. Expression Level Analysis Using Real-Time qPCR

Real-time qPCR characterized OR DEGs. Supplementary file 12: Table S8 lists the primer sequences, which were created using Beacon Designer 8.14 software (PREMIER Biosoft International, Palo Alto, CA, USA). In accordance with the manufacturer’s instructions, cDNA was synthesized using the Polestar 1st cDNA Synthesis Kit (gDNA removal) (Beijing Baoying Tonghui Biotechnology Co., Ltd., Beijing, China). On the Bio-Rad CFX96 (Bio-Rad Laboratories, Inc., Hercules, CA, USA), qRT-PCR was performed using StarLighter SYBR Green qPCR Mix (Universal) (Beijing Foreverstar Biotech Co., Ltd., Beijing, China) with the following program: initial denaturation at 95 °C for 3 min, followed by 40 cycles of 95 °C for 10 s and 60 °C for 30 s. Following the amplification program, a melting curve was constructed to confirm each primer pair’s specificity. Three biological replicates were used for each reaction, along with negative controls.

Expression stability of six candidate reference genes was assessed using the GeNorm algorithm [72], which identified actin and succinate dehydrogenase complex, subunit A (SDHA) as the most stable and suitable reference gene pair for normalization (Supplementary file 13: Table S9). Relative transcription levels were calculated using the 2^−ΔΔCt^ method. Data visualization was performed using GraphPad Prism 9.0 (GraphPad Software Inc., San Diego, CA, USA), and OR differential expression between male and female antennae were conducted with Student’s *t*-test (*α* = 0.05) in SPSS 22.0 (SPSS Inc., Chicago, IL, USA).

## 5. Conclusions

This study provides a comprehensive molecular characterization of the chemosensory repertoire in *P. solenopsis*, identifying 91 candidate genes from its antennal transcriptome. Phylogenetic analysis placed these genes within a robust evolutionary framework, revealing conserved and lineage-specific features relative to other hemipterans. A key finding was the significant male-biased expression of 18 odorant receptors, strongly implicating their role in sex pheromone reception. While this work establishes a crucial foundation and proposes a mechanistic hypothesis for pheromone detection, it also highlights the necessity for functional validation. Future research should focus on deorphanizing these candidate ORs and employing techniques like RNAi to confirm their function, which will be pivotal for advancing our understanding of mealybug chemical ecology and guiding novel pest management solutions.

## Data Availability

The raw data presented in this study are available in the NCBI Short Read Archive database PRJNA1314049 (https://www.ncbi.nlm.nih.gov/sra/PRJNA1314049, accessed on 7 November 2025).

## Supplementary Materials

The following supporting information can be downloaded from https://www.mdpi.com/article/10.3390/ijms262210901/s1.
