# Supplementary material for: Comparative Antennal Transcriptome Analysis of Phenacoccus solenopsis and Expression Profiling of Candidate Odorant Receptor Genes
Source: Int J Mol Sci. 2025 Nov 10;26(22):10901. doi: 10.3390/ijms262210901 (PMC12652395; doi:10.3390/ijms262210901)
Supplement: Supplementary file 1 [file ijms-26-10901-s001.zip › Supplementary file1 Table S1 The summary data of antennal transcriptome of Phenacoccus solenopsis adults.pdf]

Table S1. The summary data of antennal transcriptome of *Phenacoccus solenopsis* adults.

Sample	Raw reads (M)	Raw bases (Gb)	Clean reads (M)	Clean bases (Gb)	Error rate (%)	Q20 (%)	Q30 (%)	GC (%)
MA_1	37.22	5.58	35.87	5.38	0.03	94.44	89.11	32.61
MA_2	46.46	6.97	44.71	6.71	0.03	95.55	90.77	34.77
MA_3	45.96	6.89	44.57	6.69	0.03	95.18	90.23	34.18
FA_1	38.15	5.72	36.43	5.46	0.03	94.62	89.55	35.54
FA_2	46.34	6.95	44,50	6.67	0.04	93.34	87.93	34.98
FA_3	42.55	6.38	40.56	6.08	0.03	94.61	89.72	35.25
