# Supplementary material for: Comparative Antennal Transcriptome Analysis of Phenacoccus solenopsis and Expression Profiling of Candidate Odorant Receptor Genes
Source: Int J Mol Sci. 2025 Nov 10;26(22):10901. doi: 10.3390/ijms262210901 (PMC12652395; doi:10.3390/ijms262210901)
Supplement: Supplementary file 1 [file ijms-26-10901-s001.zip › Supplementary file2 Figure S1.pdf]

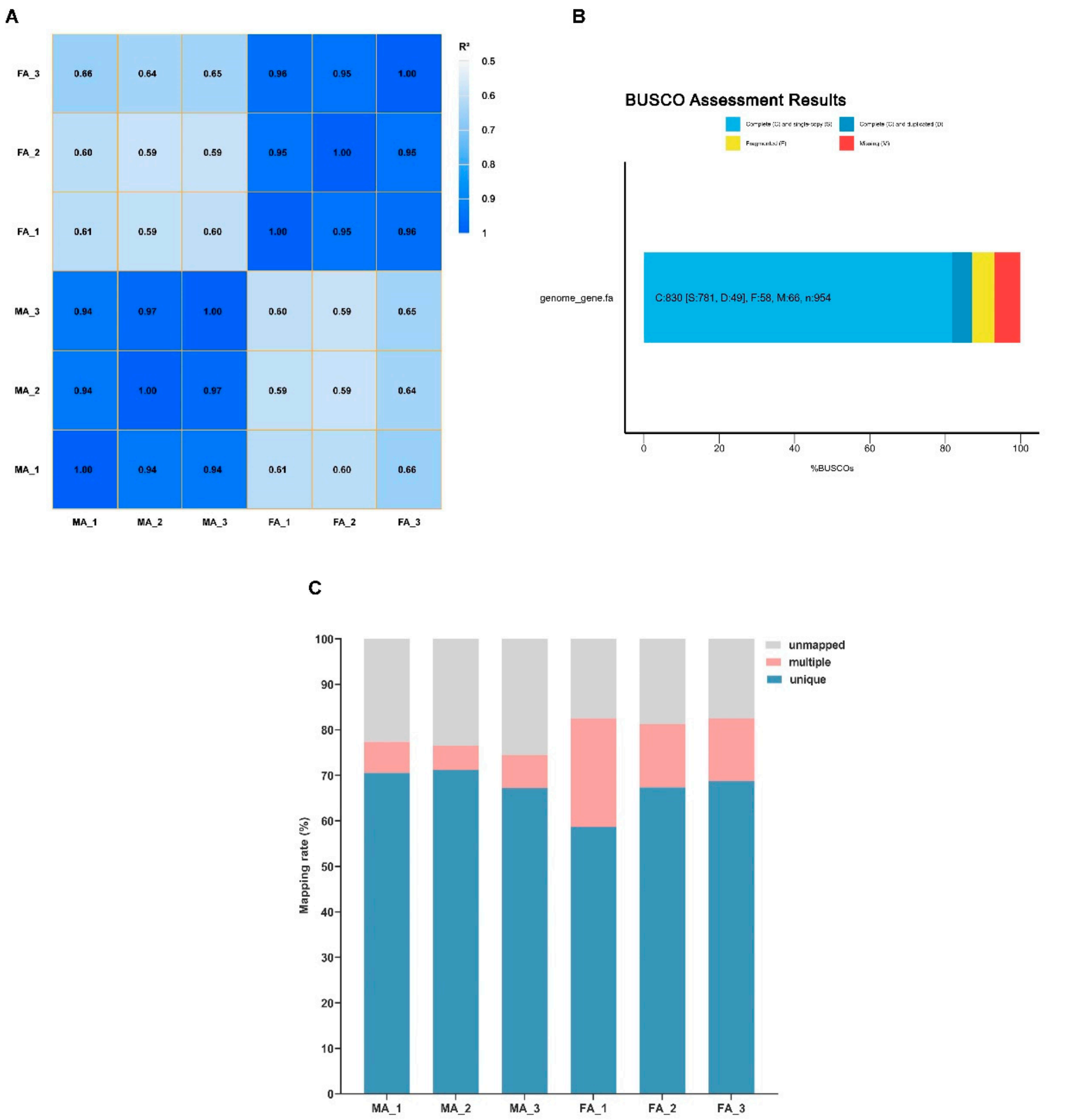

Figure S1. (A) Correlation analysis of individual biological replicates in males and females. (B) Assessment of genome assembly completeness of *Phenacoccus solenopsis* using BUSCO analysis. (C) Mapping ratio of the sample to the reference genome.
