# Supplementary material for: Comparative Antennal Transcriptome Analysis of Phenacoccus solenopsis and Expression Profiling of Candidate Odorant Receptor Genes
Source: Int J Mol Sci. 2025 Nov 10;26(22):10901. doi: 10.3390/ijms262210901 (PMC12652395; doi:10.3390/ijms262210901)
Supplement: Supplementary file 1 [file ijms-26-10901-s001.zip › Supplementary file3 Table S2 The results of unigene annotation of the antennal transcriptome of Phenacoccus solenopsis.pdf]

**Table S2.** The results of unigene annotation of the antennal transcriptome of *Phenacoccus solenopsis*.

| **Database** | **Unigenes** | **Percentage (%)** |
|---|---|---|
| Annotated in NR | 13,071 | 87.24 |
| Annotated in Swiss-Prot | 10,100 | 67.41 |
| Annotated in PFAM | 9739 | 65.00 |
| Annotated in KOG | 6560 | 43.79 |
| Annotated in GO | 6443 | 43.00 |
| Annotated in KEGG | 2979 | 19.88 |
| Not annotated in any database | 1212 | 8.09 |
| Annotated in at least one database | 13,770 | 91.91 |
