# Supplementary material for: Comparative Antennal Transcriptome Analysis of Phenacoccus solenopsis and Expression Profiling of Candidate Odorant Receptor Genes
Source: Int J Mol Sci. 2025 Nov 10;26(22):10901. doi: 10.3390/ijms262210901 (PMC12652395; doi:10.3390/ijms262210901)
Supplement: Supplementary file 1 [file ijms-26-10901-s001.zip › Supplementary file4 Figure S2.pdf]

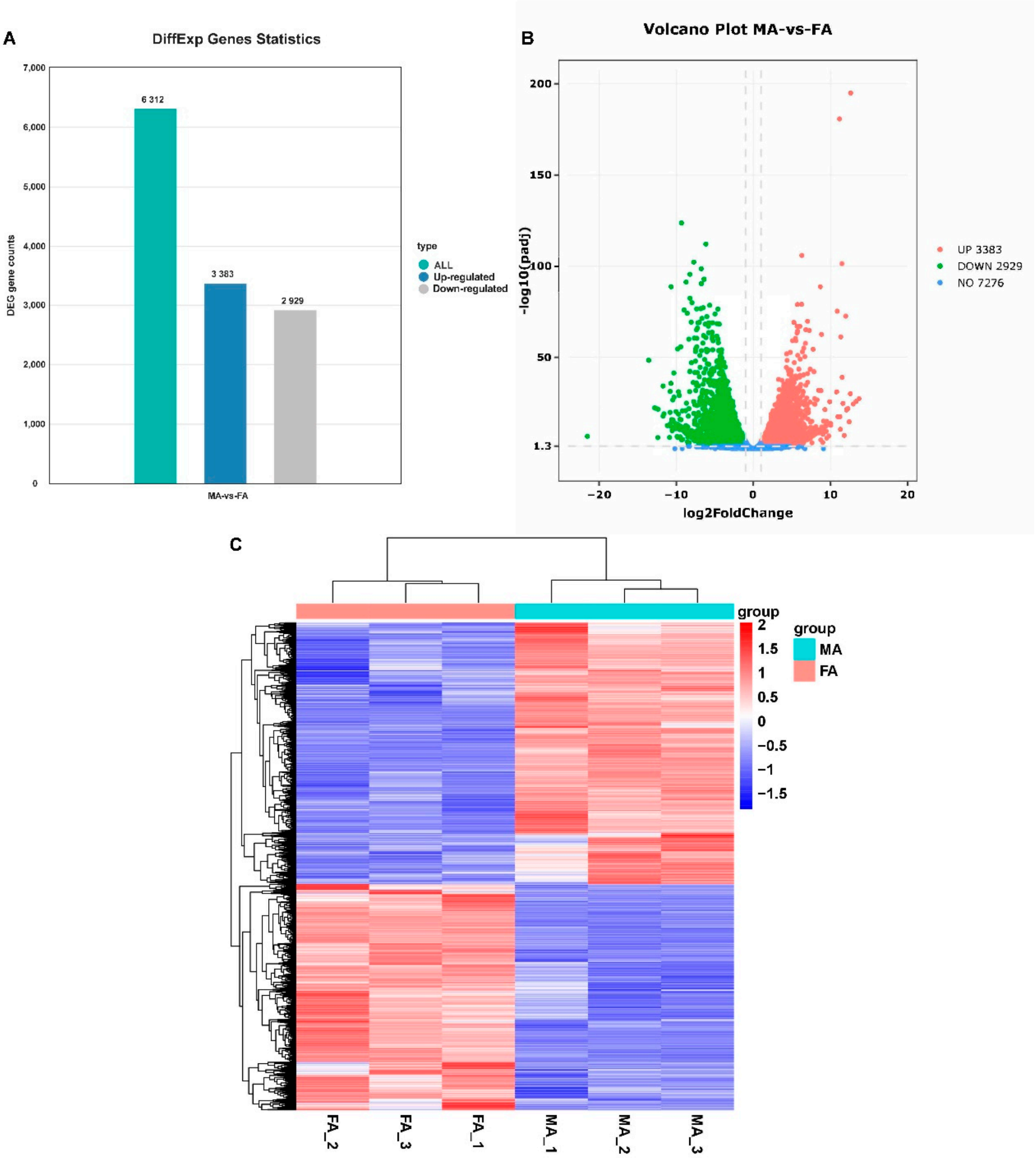

Figure S2. (A) Differential gene statistical map. (B) The volcano map of gens for sex difference of *P. solenopsis* (male vs. female). The dashed lines mean FDR < 0.05 or |log2FC| > 1. (C) DEG heat map for gene expression clustering.
