# Supplementary material for: Comparative Antennal Transcriptome Analysis of Phenacoccus solenopsis and Expression Profiling of Candidate Odorant Receptor Genes
Source: Int J Mol Sci. 2025 Nov 10;26(22):10901. doi: 10.3390/ijms262210901 (PMC12652395; doi:10.3390/ijms262210901)
Supplement: Supplementary file 1 [file ijms-26-10901-s001.zip › Supplementary file7 Table S5 Candidate Phenacoccus solenopsis antennal chemosensory genes.pdf]

Table S5-1 Identification and differential expression analysis of odorant receptors in *Phenacoccus solenopsis*

Gene name	ORF (aa)	Blastx best hit (Reference/Name/Species)	E-value	Identity (%)	TMD (No.)	Full length	FPKM (mean ± SE)		Whether differential
							MA	FA	
PsolOR1	446	ref|XP_065225221.1|odorant receptor 43a-like isoform X2 [*Planococcus citri*]	1.00E-10	26.59	6	Yes	2.92 ± 0.77	0.23 ± 0.05	Yes
PsolORco	445	gb|ANW12106.1|olfactory receptor protein 1 [*Phenacoccus solenopsis*]	0	95.49	7	Yes	426.31 ± 27.16	15.93 ± 4.12	Yes
PsolOR2	445	ref|XP_065225221.1|odorant receptor 43a-like isoform X2 [*Planococcus citri*]	2.00E-11	40.74	6	Yes	4.13 ± 0.73	0.00 ± 0.00	Yes
PsolOR3	443	ref|XP_065225220.1|odorant receptor 43a-like isoform X1 [*Planococcus citri*]	3.00E-10	28.02	6	Yes	1.90 ± 0.32	0.07 ± 0.01	Yes
PsolOR4	434	ref|XP_065225221.1|odorant receptor 43a-like isoform X2 [*Planococcus citri*]	3.00E-09	40.00	6	No	0.88 ± 0.32	0.09 ± 0.07	No
PsolOR5	431	ref|XP_065225221.1|odorant receptor 43a-like isoform X2 [*Planococcus citri*]	3.00E-09	37.35	6	Yes	3.78 ± 0.19	0.09 ± 0.02	Yes
PsolOR6	428	gb|ALV87631.1|odorant receptor 16 [*Drosicha corpulenta*]	1.00E-27	26.14	6	Yes	3.47 ± 0.58	0.64 ± 0.27	Yes
PsolOR7	427	ref|XP_065205950.1|odorant receptor 22c-like [*Planococcus citri*]	7.00E-23	33.58	6	Yes	1.56 ± 0.19	0.21 ± 0.10	Yes
PsolOR8	426	ref|XP_065215928.1|odorant receptor 4-like [*Planococcus citri*]	9.00E-54	30.33	6	Yes	9.52 ± 0.68	0.02 ± 0.02	Yes
PsolOR9	425	ref|XP_065203044.1|odorant receptor 10a-like isoform X3 [*Planococcus citri*]	4.00E-45	27.91	6	Yes	4.50 ± 1.12	0.00 ± 0.00	Yes
PsolOR10	425	ref|XP_065203044.1|odorant receptor 10a-like	3.00E-45	30.34	6	Yes	4.76 ± 0.40	0.12 ± 0.02	Yes

		isoform X3 [*Planococcus citri*]							
PsolOR11	425	ref|XP_065203044.1|odorant receptor 10a-like isoform X3 [*Planococcus citri*]	5.00E-35	27.91	6	No	3.24 ± 0.45	0.00 ± 0.00	Yes
PsolOR12	425	ref|XP_065215928.1|odorant receptor 4-like [*Planococcus citri*]	5.00E-49	29.44	6	Yes	276.50 ± 27.69	0.05 ± 0.02	Yes
PsolOR13	425	ref|XP_065203044.1|odorant receptor 10a-like isoform X3 [*Planococcus citri*]	1.00E-45	29.00	6	Yes	8.02 ± 1.78	0.00 ± 0.00	Yes
PsolOR14	425	ref|XP_065203008.1|odorant receptor Or2-like [*Planococcus citri*]	4.00E-38	28.50	6	Yes	1.54 ± 0.19	0.00 ± 0.00	Yes
PsolOR15	425	ref|XP_065203044.1|odorant receptor 10a-like isoform X3 [*Planococcus citri*]	2.00E-38	30.30	6	No	1.50 ± 0.13	0.01 ± 0.01	Yes
PsolOR16	424	gb|ALV87623.1|odorant receptor 8 [*Drosicha corpulenta*]	3.00E-41	30.13	6	No	1.69 ± 0.08	1.85 ± 0.26	No
PsolOR17	418	ref|XP_065203044.1|odorant receptor 10a-like isoform X3 [*Planococcus citri*]	2.00E-09	21.78	6	Yes	14.08 ± 1.39	0.02 ± 0.02	Yes
PsolOR18	405	gb|ALV87631.1|odorant receptor 16 [*Drosicha corpulenta*]	5.00E-06	28.28	6	Yes	0.35 ± 0.17	0.00 ± 0.00	Yes
PsolOR19	405	gb|ALV87623.1|odorant receptor 8 [*Drosicha corpulenta*]	4.00E-36	29.00	6	Yes	9.46 ± 1.71	0.00 ± 0.00	Yes
PsolOR20	404	gb|ALV87624.1|odorant receptor 9 [*Drosicha corpulenta*]	3.00E-35	27.46	6	Yes	6.73 ± 0.79	1.42 ± 0.24	Yes
PsolOR21	402	ref|XP_065223676.1|odorant receptor 85f [*Planococcus citri*]	1.00E-26	28.02	6	Yes	27.62 ± 3.46	0.28 ± 0.04	Yes
PsolOR22	394	gb|ALV87631.1|odorant receptor 16 [*Drosicha corpulenta*]	3.00E-15	27.14	6	Yes	2.07 ± 0.22	0.34 ± 0.17	Yes
PsolOR23	393	gb|ALV87623.1|odorant receptor 8 [*Drosicha*	4.00E-23	25.96	6	Yes	1.71 ± 0.12	0.00 ± 0.00	Yes

		corpulenta]							
PsolOR24	388	ref|XP_065223122.1|odorant receptor 2a [*Planococcus citri*]	6.00E-33	26.94	6	No	1.98 ± 0.28	0.38 ± 0.07	Yes
PsolOR25	383	ref|XP_065215928.1|odorant receptor 4-like [*Planococcus citri*]	4.00E-39	28.31	6	Yes	2.05 ± 0.16	0.00 ± 0.00	Yes
PsolOR26	357	ref|XP_065205950.1|odorant receptor 22c-like [*Planococcus citri*]	3.00E-22	41.84	5	No	0.02 ± 0.02	1.73 ± 0.51	Yes
PsolOR27	345	ref|XP_065225221.1|odorant receptor 43a-like isoform X2 [*Planococcus citri*]	2.00E-08	35.44	5	No	1.56 ± 0.97	0.00 ± 0.00	Yes
PsolOR28	332	ref|XP_065205950.1|odorant receptor 22c-like [*Planococcus citri*]	8.00E-15	31.88	5	No	0.97 ± 0.40	0.91 ± 0.07	No
PsolOR29	315	gb|ALV87631.1|odorant receptor 16 [*Drosicha corpulenta*]	1.00E-13	28.40	4	No	1.78 ± 0.38	0.27 ± 0.12	Yes
PsolOR30	308	ref|XP_065205950.1|odorant receptor 22c-like [*Planococcus citri*]	9.00E-08	29.41	4	No	1.65 ± 0.34	1.34 ± 0.51	No
PsolOR31	207	gb|ALV87631.1|odorant receptor 16 [*Drosicha corpulenta*]	3.00E-09	26.34	2	No	0.28 ± 0.08	1.88 ± 0.54	Yes
PsolOR32	184	gb|ALV87623.1|odorant receptor 8 [*Drosicha corpulenta*]	2.00E-22	30.54	3	No	1.54 ± 0.77	0.34 ± 0.13	No
PsolOR33	155	gb|ALV87623.1|odorant receptor 8 [*Drosicha corpulenta*]	7.00E-23	31.50	2	No	0.93 ± 0.30	0.06 ± 0.05	No
PsolOR34	117	gb|ANW12107.1|olfactory receptor protein 2 [*Phenacoccus solenopsis*]	4.00E-20	41.41	1	No	20.26 ± 2.52	3.70 ± 0.43	Yes
PsolOR35	99	gb|ALV87623.1|odorant receptor 8 [*Drosicha corpulenta*]	7.00E-14	48.04	1	No	0.58 ± 0.13	0.20 ± 0.14	No
PsolOR36	96	gb|ALV87617.1|odorant receptor 2 [*Drosicha	6.00E-13	35.05	1	No	1.24 ± 0.14	0.30 ± 0.14	No

		corpulenta]								
PsolOR37	85	ref|XP_065203008.1|odorant receptor Or2-like [*Planococcus citri*]	2.00E-15	30.22	0	No	2.87 ± 0.42	0.00 ± 0.00	Yes
PsolOR38	71	ref|XP_065205950.1|odorant receptor 22c-like [*Planococcus citri*]	5.00E-15	37.36	0	No	0.00 ± 0.00	1.13 ± 0.60	No
PsolOR39	62	gb|ALV87623.1|odorant receptor 8 [*Drosicha corpulenta*]	3.00E-07	40.30	0	No	1.65 ± 0.56	0.70 ± 0.33	No

Table S5-2 Identification and differential expression analysis of gustatory receptors in *Phenacoccus solenopsis*

Gene name	ORF (aa)	Blastx best hit (Reference/Name/Species)	E-value	Identity (%)	TMD (No.)	Full length	FPKM (mean ± SE)		Whether differential
							MA	FA	
PsolGR1	663	gb| BES90661.1|7 transmembrane sweet-taste receptor of 3 GCPR [*Nesidiocoris tenuis*]	0	67.81	7	Yes	1.05 ± 0.06	0.04 ± 0.04	Yes
PsolGR2	421	gb|BES97782.1|7 transmembrane sweet-taste receptor of 3 GCPR [*Nesidiocoris tenuis*]	1.00E-169	60.78	7	Yes	0.48 ± 0.22	0.03 ± 0.03	No
PsolGR3	420	gb|BES97782.1|7 transmembrane sweet-taste receptor of 3 GCPR [*Nesidiocoris tenuis*]	2.00E-170	61.18	7	Yes	1.68 ± 0.15	0.31 ± 0.17	Yes
PsolGR4	347	ref|XP_065221543.1|putative gustatory receptor 28b [*Planococcus citri*]	8.00E-78	92.97	6	No	0.07 ± 0.03	0.00 ± 0.00	No
PsolGR5	340	ref|XP_065215626.1|gustatory receptor for sugar taste 64f-like [*Planococcus citri*]	0	80.06	7	No	0.00 ± 0.00	0.15 ± 0.06	No
PsolGR6	331	ref|XP_065217158.1|gustatory and odorant receptor 24-like [*Planococcus citri*]	4.00E-64	37.27	7	No	0.16 ± 0.06	0.00 ± 0.00	No

PsolGR7	259	ref| XP_050058208.1|gustatory receptor for sugar taste 61a-like isoform X1 [*Aphis gossypii*]	7.00E-69	45.15	5	No	0.00 ± 0.00	0.00 ± 0.00	No
PsolGR8	240	ref| XP_065199960.1|gustatory receptor for sugar taste 64f-like [*Planococcus citri*]	8.00E-142	91.56	3	No	8.86 ± 0.41	3.54 ± 0.14	Yes
PsolGR9	234	ref|XP_050058244.1|gustatory receptor for sugar taste 61a-like isoform X2 [*Aphis gossypii*]	1.00E-62	44.21	5	No	0.00 ± 0.00	0.00 ± 0.00	No
PsolGR10	158	ref|XP_065221543.1|putative gustatory receptor 28b [*Planococcus citri*]	1.00E-97	94	2	No	0.66 ± 0.54	0.00 ± 0.00	No
PsolGR11	92	ref|XP_065221543.1|putative gustatory receptor 28b [*Planococcus citri*]	1.00E-96	93.96	2	No	0.00 ± 0.00	0.00 ± 0.00	No
PsolGR12	66	gb| EFA07621.2|gustatory receptor 125 [*Tribolium castaneum*]	1.00E-11	51.85	0	No	0.00 ± 0.00	0.00 ± 0.00	No
PsolGR13	53	gb| EFA07621.2|gustatory receptor 125 [*Tribolium castaneum*]	1.00E-08	60.87	0	No	0.00 ± 0.00	0.00 ± 0.00	No

Table S5-3 Identification and differential expression analysis of ionotropic receptors in *Phenacoccus solenopsis*

Gene name	ORF (aa)	Blastx best hit (Reference/Name/Species)	E-value	Identity (%)	TMD (No.)	Full length	FPKM (mean ± SE)		Whether differential
							MA	FA	
PsolIR40a	1611	ref|XP_065226261.1|ionotropic receptor 40a [*Planococcus citri*]	0	90.37	5	Yes	23.59 ± 2.56	35.99 ± 5.86	No
PsolIR93a	897	ref|XP_065210969.1|ionotropic receptor 93a-like isoform X1 [*Planococcus citri*]	0	81.40	4	Yes	17.27 ± 1.08	33.29 ± 2.29	No
PsolIR68a	724	ref|XP_018901146.2|ionotropic receptor 21a isoform X1 [*Bemisia tabaci*]	0	49.92	3	Yes	3.85 ± 0.48	6.77 ± 0.11	No

PsolIR25a	602	ref|XP_065220958.1|ionotropic receptor 25a [*Planococcus citri*]	0	88.09	3	No	2.94 ± 0.52	0.87 ± 0.08	Yes
PsolIR323	464	ref|XP_065200888.1|glutamate receptor ionotropic, kainate glr-3-like [*Planococcus citri*]	2.00E-110	75.00	3	Yes	13.20 ± 2.47	7.48 ± 1.13	No
PsolIR21a	412	ref|XP_065202562.1|ionotropic receptor 21a [*Planococcus citri*]	2.00E-114	57.36	0	No	3.20 ± 0.41	0.50 ± 0.24	Yes
PsolIR325	390	ref|XP_027837340.2|glutamate receptor ionotropic, delta-2 [*Aphis gossypii*]	4.00E-119	45.45	3	No	0.14 ± 0.08	2.04 ± 0.43	Yes
PsolIR100a	379	ref|XP_065219714.1|glutamate receptor ionotropic, NMDA 1-like [*Planococcus citri*]	0	84.94	2	No	0.02 ± 0.01	0.10 ± 0.08	No
PsolIR8a	261	ref|XP_065214967.1|ionotropic receptor 25a [*Planococcus citri*]	5.00E-82	59.05	2	No	0.00 ± 0.00	0.08 ± 0.07	No
PsolIR68a.1	98	ref|XP_018901146.2|ionotropic receptor 21a isoform X1 [*Bemisia tabaci*]	1.00E-15	56.18	1	No	1.96 ± 0.25	0.44 ± 0.36	No
PsolIR1	510	ref|XP_065225872.1|glutamate receptor ionotropic, kainate 2-like [*Planococcus citri*]	0	64.08	0	No	2.15 ± 0.39	0.58 ± 0.17	Yes
PsolIR2	375	ref|XP_065214406.1|adhesion G protein-coupled receptor E2-like isoform X3 [*Planococcus citri*]	4.00E-57	32.22	4	Yes	0.01 ± 0.01	0.00 ± 0.00	No
PsolIR3	278	ref|XP_065211910.1|glutamate receptor ionotropic, NMDA 2B isoform X2 [*Planococcus citri*]	5.00E-159	82.43	1	No	0.75 ± 0.08	1.16 ± 0.46	No
PsolIR4	260	gb| QPZ88978.1|ionotropic receptor 14, partial [*Diaphorina citri*]	7.00E-28	33.33	0	No	59.25 ± 7.80	16.55 ± 4.64	Yes

PsolIR5	182	gb|WHU27578.1|ionotropic receptor 1, partial [*Matsumurasca onukii*]	4.00E-72	63.93	0	No	7.28 ± 1.49	2.39 ± 0.88	Yes
PsolIR6	150	ref|XP_065215562.1|glutamic acid-rich protein-like [*Planococcus citri*]	1.00E-16	94.12	0	No	17.62 ± 1.34	68.50 ± 4.91	Yes
PsolIR7	142	ref|XP_065211909.1|glutamate receptor ionotropic, NMDA 2B isoform X1 [*Planococcus citri*]	9.00E-56	63.31	0	No	0.61 ± 0.14	0.00 ± 0.00	No
PsolIR8	128	ref|XP_072160686.1|glutamate receptor ionotropic, NMDA 2B isoform X5 [*Bemisia tabaci*]	2.00E-25	67.80	1	No	0.29 ± 0.24	1.00 ± 0.68	No
PsolIR9	106	ref|XP_065219746.1|glutamate receptor 2-like [*Planococcus citri*]	2.00E-17	53.54	1	No	4.58 ± 0.26	3.10 ± 0.32	No

Table S5-4 Identification and differential expression analysis of odorant binding proteins in *Phenacoccus solenopsis*

Gene name	ORF (aa)	Blastx best hit (Reference/Name/Species)	E-value	Identity (%)	Signal peptide	Full length	FPKM (mean ± SE)		Whether differential
							MA	FA	
PsolOBP1	199	gb|ALS31066.1|odorant-binding protein [*Phenacoccus solenopsis*]	1.00E-140	100.00	23	Yes	9.19 ± 3.17	2.66 ± 0.55	Yes
PsolOBP2	157	ref|XP_065205261.1|general odorant-binding protein 19d-like isoform X2 [*Planococcus citri*]	9.00E-70	74.15	24	Yes	19.03 ± 1.23	2.79 ± 0.27	Yes
PsolOBP3	154	gb|ALS31063.1|odorant-binding protein [*Phenacoccus solenopsis*]	2.00E-102	100.00	20	Yes	19.31 ± 2.13	726.97 ± 91.95	Yes

PsolOBP4	149	gb|ALS31054.1|odorant-binding protein [*Phenacoccus solenopsis*]	2.00E-100	100.00	20	Yes	2.33 ± 0.51	6.98 ± 1.95	Yes
PsolOBP5	147	gb|ALS31056.1|odorant-binding protein [*Phenacoccus solenopsis*]	5.00E-19	36.84	15	Yes	7925.73 ± 847.49	3.39 ± 0.76	Yes
PsolOBP6	147	gb|ALS31056.1|odorant-binding protein [*Phenacoccus solenopsis*]	2.00E-98	100.00	15	Yes	535.08 ± 26.15	220.55 ± 38.04	Yes
PsolOBP7	136	gb|ALS31057.1|odorant-binding protein [*Phenacoccus solenopsis*]	9.00E-78	85.23	No	Yes	19.81 ± 2.24	51.47 ± 5.57	Yes
PsolOBP8	135	gb|ALS31065.1|odorant-binding protein [*Phenacoccus solenopsis*]	2.00E-78	100.00	19	Yes	17210.78±3591.12	904.46±182.71	Yes
PsolOBP9	116	gb|ALS31055.1|odorant-binding protein [*Phenacoccus solenopsis*]	8.00E-93	100.00	No	No	5.30 ± 0.45	21.41 ± 1.27	Yes
PsolOBP10	107	gb|ALS31057.1|odorant-binding protein [*Phenacoccus solenopsis*]	6.00E-68	99.06	19	No	93.49 ± 3.42	710.71±120.78	Yes

Table S5-5 Identification and differential expression analysis of chemosensory proteins in *Phenacoccus solenopsis*

**Gene name**	**ORF (aa)**	**Blastx best hit (Reference/Name/Species)**	**E-value**	**Identity (%)**	**Signal peptide**	**Full length**	**FPKM (mean ± SE)**		**Whether differential**
							**MA**	**FA**	
PsolCSP1	140	gb| AJP61962.1|chemosensory protein [*Phenacoccus solenopsis*]	1.00E-82	100	28	No	9548.74 ± 473.89	448.29 ± 81.19	Yes
PsolCSP2	136	gb|AJP61956.1|chemosensory protein [*Phenacoccus solenopsis*]	5.00E-95	100	18	No	60.59 ± 15.35	30.45 ± 11.93	No
PsolCSP3	136	gb| AJP61961.1|chemosensory protein [*Phenacoccus solenopsis*]	4.00E-83	100	21	Yes	0.08 ± 0.07	8.29 ± 6.18	Yes

PsolCSP4	133	gb|AJP61951.1|chemosensory protein [*Phenacoccus solenopsis*]	1.00E-90	100	17	Yes	467.48 ± 26.06	38.26 ± 12.87	Yes
PsolCSP5	130	gb|AJP61954.1|chemosensory protein [*Phenacoccus solenopsis*]	3.00E-62	100	19	Yes	39.59 ± 7.62	3.73 ± 0.87	Yes
PsolCSP6	117	gb|AJP61960.1|chemosensory protein [*Phenacoccus solenopsis*]	2.00E-59	100	19	Yes	28429.91 ± 5515.20	5536.58 ± 1621.56	Yes
PsolCSP7	111	gb|AJP61958.1|chemosensory protein [*Phenacoccus solenopsis*]	1.00E-70	88.10	20	Yes	23509.30 ± 4446.74	24724.94 ± 7936.13	No

Table S5-6 Identification and differential expression analysis of sensory neuron membrane proteins in *Phenacoccus solenopsis*

Gene name	ORF (aa)	Blastx best hit (Reference/Name/Species)	E-value	Identity (%)	TMD (No.)	Full length	FPKM (mean ± SE)		Whether differential
							MA	FA	
PsolSNMP1	474	ref|XP_065212463.1|sensory neuron membrane protein 1 [*Planococcus citri*]	0	81.36	1	Yes	610.34 ± 54.52	19.06 ± 4.57	Yes
PsolSNM2	506	ref|XP_065206241.1|scavenger receptor class B member 1 isoform X2 [*Planococcus citri*]	0	83.83	1	Yes	52.87 ± 11.28	7.98 ± 2.33	Yes
