# Supplementary material for: Comparative Antennal Transcriptome Analysis of Phenacoccus solenopsis and Expression Profiling of Candidate Odorant Receptor Genes
Source: Int J Mol Sci. 2025 Nov 10;26(22):10901. doi: 10.3390/ijms262210901 (PMC12652395; doi:10.3390/ijms262210901)
Supplement: Supplementary file 1 [file ijms-26-10901-s001.zip › Supplementary file9 Figure S3 Amino acid sequence identities of the classic odorant binding proteins from Phenacoccus solenopsis.pdf]

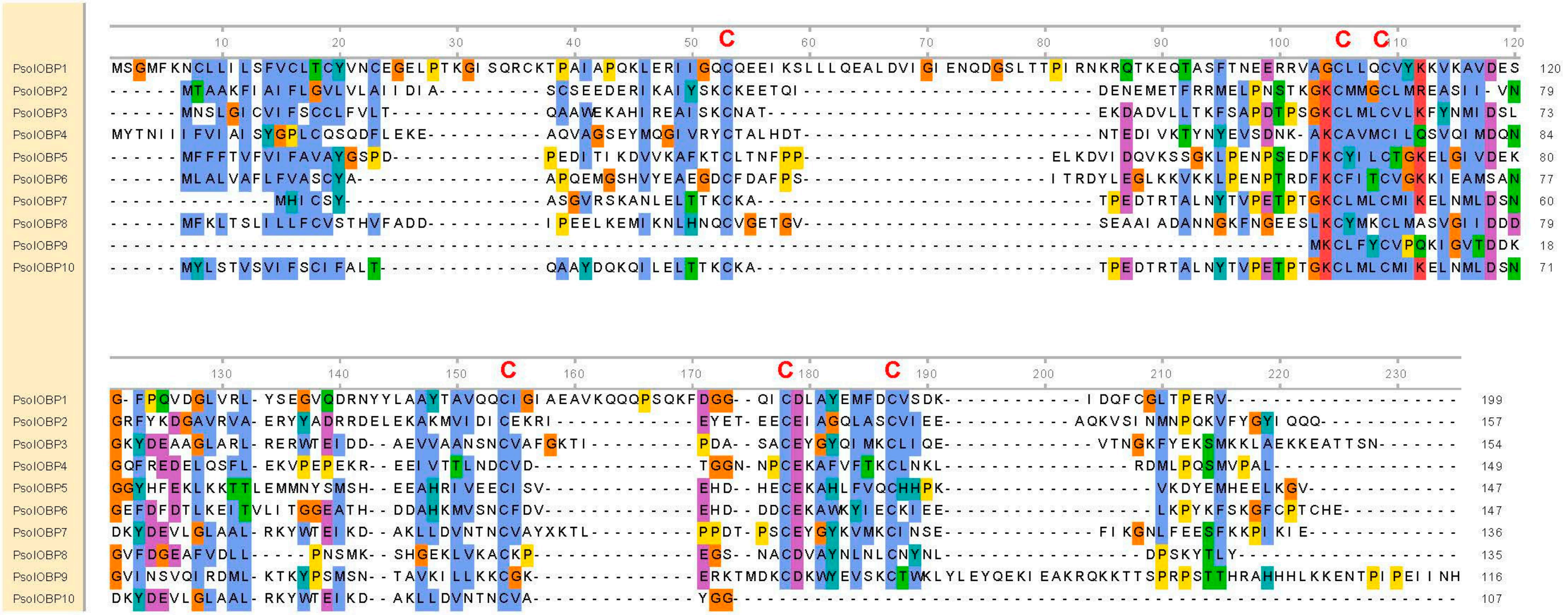

Figure S3. Amino acid sequence identities of the Classic odorant binding proteins (OBPs) from *Phenacoccus solenopsis.* The position of the conserved cysteine residues of OBPs from *P. solenopsis* are marked by bold red letters "C".
