# Supplementary material for: Comparative Antennal Transcriptome Analysis of Phenacoccus solenopsis and Expression Profiling of Candidate Odorant Receptor Genes
Source: Int J Mol Sci. 2025 Nov 10;26(22):10901. doi: 10.3390/ijms262210901 (PMC12652395; doi:10.3390/ijms262210901)
Supplement: Supplementary file 1 [file ijms-26-10901-s001.zip › Supplementary file12 Table S8 List of primers used in real-time PCR for PsolORs.pdf]

Table S8 List of primers used in real-time PCR for *PsolORs*

Gene Name	Primer F	Primer R	Amplicon size (bp)	Melt temperature (°C)
**PsolOR1**	TCCTTGAAGCGTGTTGTTCA	CATCTTCTGGCACCATTCCT	219	76.5
**PsolORco**	TCACCCTGACTCTATTGGCG	GTATGCAGCTTCCATGACCG	167	79.0
**PsolOR2**	TTATTTGCCTCCGACCCAAC	TCGCCTCCATAACTCGAAGT	167	77.0
**PsolOR3**	CAACAACCCGAGACAAAGCA	TGTGACTGGCGTATCATTCG	186	75.5
**PsolOR5**	CTATTGGCGCGAAAGTCTCC	CCAGCTAACCTCCTTCGGAA	241	76.0
**PsolOR6**	TGGTACCATCGTGTATCCCA	ATCATGCAACCCACTACCGA	183	79.0
**PsolOR7**	CGGCGAATTTAATGACGGGT	ACCGCATACATGTCAGGACT	183	77.5
**PsolOR8**	CGCGAGAAGATGGCTATTGA	GCCAGCTCGCAAATAAACAT	155	77.0
**PsolOR9**	GGTTCCACTTCCGTATCT	TTCGTCGTGACATATCGTA	206	78.0
**PsolOR10**	TGATTGTCAAACGCAACACTT	CCCACGTTGCACACTAGAAA	180	77.0
**PsolOR11**	TGTTTCCACTACCGCATCTC	CGCTTCGTCGTGACATATCG	155	77.5
**PsolOR12**	TGGAAGAGTATGGCACCAGG	TAATCCTTTGACGCGATGGC	178	76.5
**PsolOR13**	TGATTGTCAAACGCAACACTT	GGCCATAGATGTGTTAGCGC	220	77.5
**PsolOR14**	TGTTTCCACTACCGCATCTC	CGCTTCGTCGTGACATATGG	155	77.5
**PsolOR15**	ACCAGAGCACCCAAGTCAAT	AGTAGATCGCTTGTGTCACCA	181	76.0
**PsolOR17**	TTTCGCGGAAGTCACGTTTT	GCCGACGTTGCAGTAAGTAA	163	76.0
**PsolOR18**	ATGCTGCCAGTAGAGTTT	AACGAGATTCACGACAGA	150	76.5
**PsolOR19**	CTTGCAGACTCGTTTCAGGT	GTGCTCGTCAGGATAGGTGT	230	76.5
**PsolOR20**	GCCTCTACGATGTTGGTTGG	ATGATGTTCGAACGACCCCA	153	77.0
**PsolOR21**	CGCAACATTTTACCGGACGA	GACATCAGACAAGGCAGCTG	159	78.0
**PsolOR22**	CATTCGGTCTTGACCATCGG	ACCTTCCAATCTGTCGCAAA	171	78.5
**PsolOR23**	CAGCGTTTCCACTACTTCTTGA	ACGCGATGAAAGCCTGAATG	175	78.5

**PsolOR24**	CAGTTGGAGGTGTTCGTGTAC	CTTTCCAACGCAGCAGTCTT	185	78.0
**PsolOR25**	AATCTACACCACGGAGTATT	ATCGCTTTCATCACCATAAC	124	77.0
**PsolOR26**	TCACATGGTACAGTCTAAGT	AACACAACGAAGGTATGAAC	75	75.0
**PsolOR27**	ACCCGTTTCCGTTTGCTAAA	CGCTGATTTTGATTCCGAGC	191	77.5
**PsolOR29**	GTGTGTTGAGTGATCGCGAA	GCAGCCACAAAAGCATAGGT	154	76.5
**PsolOR31**	GACAAGTGTAAACTATTGGCAGAGG	CGAGTAACATCAATAGCGAACG	167	77.0
**PsolOR34**	CAACCAGCACCACGAAGGA	ATGAGAAAGCCAAAAAGACGTATT	80	75.0
**PsolOR37**	CGTGGGGCAGACATTGAATG	ACGGACGATAACTTCACACAA	146	78.0
**Actin**	CAAGTTCGACATTCCCGGTG	AAGGTTTCGAGTCGCTGGT	167	78.5
**SDHA**	ATTCAGGCACCTCAAAACCG	TTCCACCAATACGAAGGGCA	250	78.5
