# Supplementary material for: Comparative Antennal Transcriptome Analysis of Phenacoccus solenopsis and Expression Profiling of Candidate Odorant Receptor Genes
Source: Int J Mol Sci. 2025 Nov 10;26(22):10901. doi: 10.3390/ijms262210901 (PMC12652395; doi:10.3390/ijms262210901)
Supplement: Supplementary file 1 [file ijms-26-10901-s001.zip › Supplementary file13 Table S9 Genorm results for selecting reference genes.pdf]

Table S9 Genorm results for selecting reference genes

Gene name	Stability value
Actin/succinate dehydrogenase complex, subunit A (*SDHA*)	1.348*
Glutathione S-transferase (*GST*)	1.457
Succinate dehydrogenase flavoprotein subunit (*SDFS*)	1.575
Ribosomal protein 28 (*28s*)	1.977
Elongation facto*r* (*EF*)	2.749

Note: The asterisk (*) indicates that these specific genes were selected as the reference genes.
